# Prevalence and predictors of long COVID at 1 year in a cohort of hospitalized patients: A multicentric qualitative and quantitative study

**DOI:** 10.1371/journal.pone.0320643

**Published:** 2025-04-11

**Authors:** Vignesh Kumar J., Jency Maria Koshy, Divyashree S., Suneetha Narreddy, Mahasampath Gowri S., Priscilla Rupali, Sowmya Sathyendra

**Affiliations:** 1 Department of Infectious Diseases, Christian Medical College, Vellore, Tamil Nadu, India; 2 Depatment of General Medicine, Believers Church Medical College Hospital, Kerala, India; 3 Infectious Diseases, MGM New Bombay Hospital, Navi Mumbai, Maharashtra, India; 4 Infectious Diseases, Apollo Health City, Hyderabad, Telangana, India; 5 Department of Biostatistics, Christian Medical College, Vellore, Tamil Nadu, India; 6 Department of Medicine-III, Christian Medical College, Vellore, Tamil Nadu, India; Hamdard Institute of Medical Science and Research, INDIA

## Abstract

**Introduction:**

According to WHO long COVID is defined as a continuation or development of new symptoms 3 months after the initial SARS-CoV-2 infection, with these symptoms lasting for at least 2 months with no other explanation. We followed up patients after an episode of acute COVID-19 for 1 year after hospital discharge from different parts of India.

**Methods:**

This was a multi-centric study among patients ≥18 years hospitalized with COVID-19, which recruited patients at 6 weeks after hospital discharge (baseline). Quantitative data on demographics, pre-existing co-morbidities, risk factors, signs and symptoms and hospital parameters during acute COVID-19 infection were noted at baseline. They were followed up and data collected telephonically thereafter at 3–6, 6–9 and 9–12 months regarding self-reported persistence of symptoms. A qualitative component included face to face in-depth interviews to elicit information on perceived health problems, quality of life and financial burden due to COVID-19.

**Results:**

A total of 315 patients were enrolled, with the majority being males (59.4%). The median age was 52 years (IQR 40, 63). The prevalence of long COVID was 16.5%. At 6 weeks and 12 months, 35.2% and 25.9% of patients, reported more than one new symptom that affected their quality of life. Shortness of breath was common at each time point, persistent muscle pain and weakness waxed and waned. Variables at 6 weeks post discharge (baseline) such as shortness of breath (OR 2.22 CI 1.06–4.65, p = <0.05), cough (OR 6.93, CI 2.36–20.30, p = <0.05), fatigue (OR 2.34, CI 1.05–5.23, p = <0.05), and weight loss (OR 2.90, CI 1.30–6.49, p = <0.05) were significantly associated with long COVID.

**Conclusion:**

We found that long COVID was noted in 16.5% patients who self reported non – recovery at 1 year. Physical symptoms, mental health issues and mobility were persistent in a significant number of patients following an initial recovery from an acute COVID-19 infection. In 25.9% of patients more than 1 symptom was reported at 1 year after COVID-19. We urgently need therapeutic interventions which can improve the quality of life in these patients.

## Introduction

COVID-19 infection has been a global pandemic with devastating medical, financial, and societal implications, many of which are still felt today. The World Health Organization (WHO) has defined post COVID 19 condition or long COVID as a continuation or development of new symptoms 3 months after the initial SARS-CoV-2 infection, with these symptoms lasting for at least 2 months with no other explanation [1].

Some estimates indicate that 10% of those infected with COVID-19 would likely have long COVID at 6 months [2,3]. The incidence of long COVID seems to depend on the severity of the acute COVID illness. It varies from 50–70% among those hospitalized to 10–30% in those not hospitalized. However, these estimates are all from high income and upper middle-income countries [4]. Incidence of long COVID was noted to be higher in pre-Omicron variants causing infection of almost up to 35% in a dataset looking at developing a definition of long COVID.[2]

In India, the prevalence has varied from 30.34% - 37.3% with data collected among health care workers via different symptom checklists or patient health questionnaires using different methodologies and definitions [5,6].

A single-center study in Eastern India suggested that the proportion of long COVID was 29% during the Delta wave and 8% in individuals infected with the Omicron variant. However, this study had a maximum follow-up of only up to 6 months [7]. Other studies from North India reported a high incidence of long COVID, however these studies had varying periods of short follow up ranging from 4–16 weeks with definitions inconsistent with the widely accepted WHO clinical case definition of long COVID where the symptoms should have lasted greater than 2 months without an alternative diagnosis [1,8–10].

Acute symptoms in COVID-19 can be wide and varied and can often persist. Long COVID can affect virtually any organ system in the body. Common clinical manifestations involve cardiac (e.g., chest pain, palpitations), pulmonary (cough, dyspnea), and gastrointestinal systems. Central nervous system manifestations can include cognitive impairment, fatigue, disordered sleep, memory loss and tinnitus due to dysautonomia, postural orthostatic tachycardia syndrome (POTS) attributed to neuroinflammation and reduced cerebral blood flow [11]. Other important clinical manifestations reported include hematological abnormalities like coagulopathy, thromboembolic phenomena (stroke and pulmonary embolism), endothelial dysfunction and microangiopathy [12] and multi-system manifestations like myalgic encephalitis/chronic fatigue syndrome. Mental health (anxiety, depression, sleep disturbance) and musculoskeletal problems, fatigue [13] have also emerged as important components of long COVID. Recent reports suggest that some patients who recover from an acute episode of COVID-19 infection, may continue to be affected by hypoxia and have persistent symptoms like dyspnea, reduced ability to work, with 11%-24% of COVID-19 patients experiencing long-term symptoms even three months beyond the onset of COVID-19 illness [13–15].

There is considerable overlap between the risk factors for long COVID from those described for severe acute COVID-19 infection, though in 30% of cases, no obvious risk factor was identified [18–20]. Common risk factors for long COVID described thus far in literature include middle age, female sex, diabetes mellitus, connective tissue disorders, attention deficit hyperactivity disorders, chronic urticaria and allergic rhinitis and presence of autoantibodies [19,21].

We therefore conducted a multi-center study in Southern and Western India including centers in Mumbai, Vellore, Kerala and Hyderabad to explore the incidence, clinical presentation and risk factors of long COVID at 1 year in patients hospitalized with different severities of acute COVID-19 infection using a modified WHO clinical case definition of long COVID or post COVID 19 condition [14].

Though many studies in India have been able to quantify persistence of various symptoms as long COVID, many of the mental health challenges have not been captured. Apart from quantitatively defining possible clinical variables, we also set out to capture a qualitative aspect of long COVID looking into various social and economic factors which have not been assigned due importance in literature thus far.

## Materials and methods

This multi-centric observational cohort study was conducted at four tertiary care hospitals in Western and Southern India assessing the incidence and clinical manifestations of long COVID in patients hospitalized for acute COVID-19. Patients were recruited at six weeks post hospital discharge (baseline) and followed up at specified intervals from 7/06/2021 to 30/09/2022.

### Inclusion and exclusion criteria

Patients aged 18 years and older, at ≥ 6 weeks after hospital discharge (baseline) from an acute episode of COVID-19 (laboratory and physician-confirmed) were recruited and all data regarding co-morbidities and symptoms were collected telephonically at baseline and at 3–6 months, 6–9 months and 9–12 months. We chose to recruit patients at ≥ 6 weeks after hospital discharge to ensure that subjective symptoms like breathlessness and fatigue were not due to the acute episode. We excluded patients with existing severe cognitive or neurodegenerative disease at baseline, patients who were unable to speak or those for whom no secondary contact person or guardian were available.

Telephonic consent was obtained from the participants prior to enrollment into the study. This study was approved by the Institutional Review Board, CMC (IRB No: 13853, dt. 24-03-2021) and ethics committees at the participating centers. This study was conducted in four hospitals namely, i) Christian Medical College, Vellore, ii) MGM New Bombay Hospital, Navi Mumbai, iii) Apollo Health City, Hyderabad and iv) Believers Church Medical College, Hospital, Thiruvalla, Kerala. The recruitment period for each of these hospitals were 20/06/2021, 17/08/2021,30/08/2021 and 7/06/2021 respectively and the end dates for each of these hospitals were 30/09/2022, 19/09/2022, 12/08/2022 and 16/06/2022 respectively

### Definition

We modified our definition of long COVID (post COVID-19 condition) from the WHO case definition [14] as follows: Persistence of self-reported overall non-recovery at 12 months after hospital discharge for the initial SARS-CoV-2 infection. Non recovery as perceived by patients could include any persistent physical or mental health symptom or sign that impacted their quality of life.

### Quantitative variables

Variables collected at study baseline included demographic data, co-morbidities, history of ICU admission, history of prolonged hospital stay during acute COVID-19 infection, re-admissions, history of complications during previous COVID-19 illness including; deep vein thrombosis (DVT), pulmonary embolism and recent febrile illness symptoms during hospitalization. Patient reported symptoms were collected via a symptom checklist at baseline (6 weeks from hospital discharge) and at intervals of 3–6 months, 6–9 months and 9–12 months up to 1 year of follow up. Standardized surveys (ISARIC Global COVID-19 follow up study protocol v. 1.0 17 Nov. 2020) [22] were used to document new or persistent symptoms and quality of life variables at pre-defined timepoints [9]. Quality of Life Scale was adopted from ISARIC (ISARIC Global COVID-19 follow up study protocol v. 1.0 17 Nov. 2020) and EQ-5D-5L, dyspnea (assessed using MRC dyspnea scale) [22]. Difficulties in functioning (UN/Washington disability score), lifestyle and socioeconomic data were also collected. Patients in all categories of severity of acute COVID, i.e., mild, moderate and severe were enrolled to provide an accurate picture of long COVID that ensued [23].

### Qualitative variables

Qualitative aspects of long COVID were captured by face to face interviews conducted by a qualified physician/social worker. Open-ended interview format was used to elicit information on health problems, quality of life, and financial burden due to COVID-19. An interview guide that started with a few open-ended questions with some follow-up probes to elicit patients’ unprompted feedback regarding their psychosocial and financial issues due to COVID-19 was used. All qualitative/semi-structured interviews were audio-recorded after obtaining informed consent. All the recorded interviews were transcribed and translated into English and analysed using DEDOOSE software (SocioCultural Research Consultants, LLC). A copy of the questionnaire used is attached in the supplementary material. A COREQ (COnsolidated criteria for REporting Qualitative research) format was used to record outcomes (Table S6 in S1 File).

### Sample size

We calculated the sample size based on the proportion of two symptoms seen in long COVID depression (4.3%) and somnipathy (17.7%) provided in a previous study [20]. With a precision varying from 2–5% and desisted confidence interval of 95%, we calculated the sample to be a minimum of 224 (based on precision of 5% and proportion of 17% for somnipathy) and maximum of 395 (based on precision of 2% and expected proportion of 4.3% for depression). Hence, we aimed to reach a total sample size of at least 395, i.e., collect at least 100 participants from each collaborating hospital.

### Statistical analysis

Data were summarized using Mean (SD)/Median (IQR) for continuous variables depending on normality, and the normality was assessed using histograms and p-p plots. Categorical data were expressed as frequency along with percentages. The continuous variables among the recovery status were analyzed using a t-test or Mann-Whitney U test based on normality. ANOVA/Kruskal Wallis test was used to compare across the severity categories of COVID.

For the univariate analysis, we looked for association of the following variables with presence of “patient reported Long COVID or Post COVID-19 condition” at 1 year as per definition of Long COVID provided above. Variables gathered at study baseline (6 weeks after hospital discharge from acute COVID-19) included demographic data, co-morbidities, history of ICU admission, history of prolonged hospital stay (< 7 ≥ days) during the acute episode, as well as persisting symptoms. Categorical associations were done using the chi-square test. We performed two separate models for symptoms and hospital details adjusted for demographic variables. Two multivariate logistic regression models were performed using stepwise backward approach with p-value of 0.20 to select the important determinants of outcome. Stepwise logistic regression, keeping p-value<0.20, was used to determine the predictors of patient reported non-recovery (Long COVID) at 1 year. Collinearity was assessed during regression and VIF <5 was taken as indication of absence. The statistical significance was maintained at a 5% level for all the comparisons. All the analyses were done using, SPSS 19.0 and STATA/BE 18.0.

## Results

We recruited 315 patients from 4 hospitals at study baseline (6 weeks after discharge from acute COVID-19 infection requiring hospitalization) and followed them up for a year. Among the four tertiary care centers, the numbers recruited were - Christian Medical College, Vellore 141 (44.8%), followed by Believers Church Medical College Hospital, Kerala 88 (27.9%), New Bombay Hospital, Mumbai 62 (19.7%) and Apollo Health City, Hyderabad 24 (7.6%) (details in Supplementary Table S1 in S1 File). The results of qualitative data presented in Table 3.Of these 315 participants, 59.4% were male, and 40.6% were female. The median age was 52 years (IQR 40, 63). Among those recruited, the most common co-morbidity was diabetes mellitus (38.7%) followed by hypertension (36.8%), cardiac disease (11.7%), and chronic pulmonary disease (5.7%). The baseline characteristics, co-morbidities, and clinical severity of illness of the study cohort are described in Supplementary Table S2 in S1 File.

**Fig 1:**
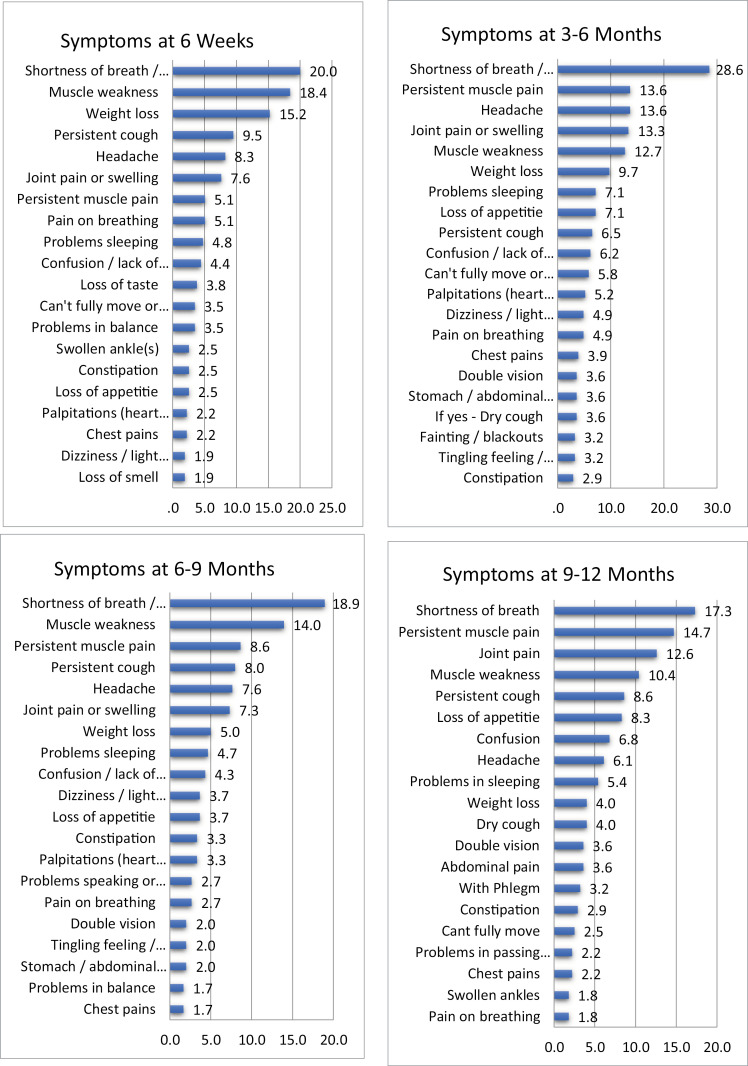
Symptoms of long COVID (each symptom represented as a % of those available for evaluation at each time point).

The overall prevalence of long COVID was 16.5% at 1 year of hospital discharge from acute COVID-19 (i.e., patient reported: “non-recovery” as specified in the methods). However, 25.9% of patients continued to have more than 1 symptom at one year. At study baseline, 35.2% patients had more than one symptom and this sequentially reduced to 31.5% at 3–6 months, 26.6% at 6–9 months and 25.9% at 9–12 months (Supplementary Table S3 in S1 File). Shortness of breath was the most common symptom at each time point, i.e., study baseline, 3 months, 6 months and 1 year. Persistent muscle pain was prominent at 3, 6, and 12 months. Overall the symptoms that were seen in more than 10% of the patients at 3, 6 or 12 months included shortness of breath, muscle weakness, persistent muscle pain, joint pain and swelling, and headache. However, muscle weakness and cough varied in frequency over time (Figure 1). Weight loss observed initially improved and pain on breathing decreased with follow-up visits.

At one year of follow up, using the EQ 5D 5L quality of life measurement survey, nearly 19.8% of patients had problems with mobility (ranging from slight to severe) and 16.9% reported problems performing their usual activities for some reason or the other, 5.8% were slightly anxious or depressed, 12.6% reported inability to fully take care of themselves and 16.9% experienced slight pain or discomfort.

A univariate logistic regression showed that persistence of shortness of breath (OR 3.56, CI 1.87–6.79, p = <0.05), cough (OR 4.08, CI 1.82–9.11, p = <0.05), joint pain (OR 3.46, CI 1.42–8.40) and weight loss (OR 3.20 CI 1.60–6.43, p = <0.05) at study baseline was associated with long COVID at 1 year. A history of ICU admission (OR 4.27 CI 2.27–8.0, p = <0.05) or prolonged hospital stay >7 days (OR 3.12 CI1.65–5.92, p = <0.05) during acute episode of COVID-19 were also found to be significantly associated with a diagnosis of long COVID at 1 year.

The multivariate analysis of symptoms adjusted for demographical variables showed that persistent shortness of breath (OR 2.88 CI 1.38–5.97, p<0.05), cough (OR 6.92, CI 2.47–19.39, p <0.001), weight loss (OR 2.79, CI 1.28–6.09, p <0.05) and joint pain (OR 3.02, CI 1.12–8.08, p <0.05) even 6 weeks after hospital discharge for acute COVID-19 (study baseline) were predictors of a diagnosis of long COVID at 1 year (Ref Table 1). Similarly a multivariate model adjusted for demographical variables revealed that a history of ICU admission (OR 2.95, CI 1.50–5.81, p = <0.05) and prolonged hospital stay > 7 days (OR 2.23 CI 1.12–4.47, p = <0.05) were predictors of long COVID at 1 year.

**Table 1:** Univariable and multivariable binary logistic regression analysis of predictors of long COVID.

Variables at study baseline or recruitment	Long COVID-19 at 1 year			Univariable Logistic Regression		Multivariable Logistic Regression of symptoms adjusted for demographic variables		Multivariable Logistic Regression of prolonged hospital stay and ICU admission adjusted for demographic variables	
	No Long COVID (N = 263)	Long COVID (N = 52)	Total (N = 315)	Odds Ratio	p-value	Adjusted Odds Ratio (95% CI)	p-value	Adjusted Odds Ratio (95% CI)	p-value
Age	179 (81.7)	40 (18.3)	219 (100)	1.56 (0.78-3.13)	0.207	0.68 (0.32–1.46)	0.330	0.75 (0.36-1.56)	0.441
Gender	159 (85)	25 (13.4)	187 (100)	0.76 (0.41-1.38)	0.376	1.02 (0.39–2.64)	0.960	1.45 (0.54–3.87)	0.461
Education	136 (83.4)	27 (16.6)	163 (100)	1.46 (0.83-2.55)	0.181	0.83 (0.40–1.72)	0.633	0.85 (0.44–1.66)	0.645
Occupation	174 (84.9)	31 (15.1)	205 (100)	0.75 (0.41-1.38)	0.367	0.711 (0.25–1.98)	0.516	1.06 (0.37 -3.02)	0.915
**Persistence of symptoms at baseline**									
Shortness of Breath	42 (66.7)	21 (33.3)	63 (100.0)	3.56 (1.87-6.79)	< 0.001	2.88 (1.38–5.97)	0.004	NA	NA
Cough	18 (60.0)	12 (40.0)	30 (100.0)	4.08 (1.82-9.11)	< 0.001	6.92 (2.47–19.39)	< 0.001	NA	NA
Weight Loss	32 (66.7)	16 (33.3)	48 (100.0)	3.20 (1.60-6.43)	< 0.001	2.79 (1.28–6.09)	0.010	NA	NA
Joint Pain	15 (62.5)	9 (37.5)	24 (100)	3.46 (1.42–8.40)	0.006	3.02 (1.12–8.08)	0.028	NA	NA
			**Variables during hospitalization of acute COVID-19**						
History of ICU admission	56 (67.5)	27 (32.5)	83 (100.0)	4.27 (2.27–8.0)	< 0.001	NA	NA	2.95 (1.50 -5.81)	0.002
History of prolonged hospital Stay (> 7 days)	110 (75.3)	36 (24.7)	146 (100.0)	3.12 (1.65-5.92)	<0.001	NA	NA	2.23 (1.12 -4.47)	0.023

Note: Both models adjusted for or controlled for demographic variables (Adjusted effect is the controlled effect of predictors assuming an equal demographic distribution)

We also administered a Quality of Life (QoL) questionnaire which indicated that even though symptoms persisted at 9–12 months, most were mobile (80.2%), able to carry out usual activities including personal care (> 80%), were not anxious or depressed (94.2%) and pain free (83%) (Table 2). QoL questionnaire stratified as per category of severity is given in supplementary Table S4 in S1 File.

**Table 2:** Best describes your health – Quality of Life – EQ 5D 5L.

Health	Severity of Problems	Before COVID (N=315)	6 Weeks (N=315)	3-6 Months (N=308)	6-9 Months (N=301)	9-12 Months (N=278)
Mobility	No problem	272 (86.3)	218 (69.2)	208 (67.5)	238 (79.1)	223 (80.2)
	Slight problems	29 (9.2)	61 (19.4)	85 (27.6)	59 (19.6)	40 (14.4)
	Moderate problems	10 (3.2)	29 (9.2)	12 (3.9)	3 (1)	10 (3.6)
	Severe problems	3 (1.0)	6 (1.9)	2 (0.6)	1 (0.3)	5 (1.8)
	Unable to walk	1 (0.3)	1 (0.3)	1 (0.3)	0 (0.0)	0 (0.0)
Usual Activities	No problem	281 (89.2)	241 (76.5)	254 (82.5)	265 (88)	231 (83.1)
	Slight problems	25 (7.9)	51 (16.2)	42 (13.6)	33 (11)	33 (11.9)
	Moderate problems	7 (2.2)	16 (5.1)	8 (2.6)	2 (0.7)	12 (4.3)
	Severe problems	1 (0.3)	6 (1.9)	2 (0.6)	1 (0.3)	2 (0.7)
	Unable to do	1 (0.3)	1 (0.3)	2 (0.6)	0 (0.0)	0 (0.0)
Anxiety/ Depression	Not anxious or depressed	303 (96.2)	263 (83.5)	274 (89)	270 (89.7)	262 (94.2)
	Slightly anxious or depressed	6 (1.9)	37 (11.7)	24 (7.8)	28 (9.3)	13 (4.7)
	Moderately anxious or depressed	4 (1.3)	14 (4.4)	7 (2.3)	3 (1)	3 (1.1)
	Severely anxious or depressed	2 (0.6)	1 (0.3)	3 (1)	0 (0.0)	0 (0.0)
	Extremely anxious or depressed	0 (0.0)	0 (0.0)	0 (0.0)	0 (0.0)	0 (0.0)
Self-Care	No problem	290 (92.1)	264 (83.8)	271 (88)	282 (93.7)	243 (87.4)
	Slight problems	16 (5.1)	33 (10.5)	24 (7.8)	17 (5.6)	22 (7.9)
	Moderate problems	5 (1.6)	12 (3.8)	10 (3.2)	1 (0.3)	10 (3.6)
	Severe problems	1 (0.3)	3 (1.0)	3 (1)	1 (0.3)	3 (1.1)
	Unable to wash or dress myself	3 (1.0)	3 (1.0)	0 (0.0)	0 (0.0)	0 (0.0)
Pain/ Discomfort	No pain or discomfort	298 (94.6)	262 (83.2)	245 (79.5)	261 (86.7)	231 (83.1)
	Slight pain or discomfort	11 (3.5)	36 (11.4)	50 (16.2)	40 (13.3)	35 (12.6)
	Moderate pain or discomfort	4 (1.3)	13 (4.1)	11 (3.6)	0 (0.0)	11 (4.0)
	Severe pain or discomfort	2 (0.6)	4 (1.3)	1 (0.3)	0 (0.0)	1 (0.4)
	Extreme pain or discomfort	0 (0.0)	0 (0.0)	1 (0.3)	0 (0.0)	0 (0.0)

**Table 3:** Challenges faced due to COVID.

Themes	Excerpts
Health Problems	*“Before COVID, I was enjoying my life, Now, I am unable to walk for a long distance. When I climb steps, I get breathing difficulties. After 10 minutes it will be alright. I am unable to walk fast like I walked previously”* (43 Years/ Male).
	*“When I inhale, I get chest pain. I have breathing difficulties. I am having tension without any reason, don’t know why. I have 10% difficulties in remembering. I am confused and often I get tense”* (57 Years/ Male)
Stigma and Discrimination	*“My neighbours avoided me. No one from my surrounding house visited me. No one told about this to me when I was hospitalized and after I was discharged I came to know this. They have a fear that COVID may spread to them. Whoever it may be there is a fear of getting COVID…”* (33/ Male)
	*“…they (the corporation staff) sprayed bleaching powder near my house and gave me some tablets in my house. All my neighbours saw this and so they avoided us. Later they keep their distance while talking to us”* (33/ Male)
	*“I have a few regular customers, but they are not coming now. I don’t know why? they did not come because I was not in the shop or because I got COVID. They talked over the phone, though they knew that I was in the shop and, they are not coming. A few customers come for purchase, but not all the regular customers are coming. Because of my absence, I lost many customers. So, I lost my business.” (Bakery business).*
Financial Burden due to hospitalization	*“I pledged my home and property and I borrowed money, my sister also helped, I pledged my wife jewels. Overall, I spend 10 Lakhs, 5 Lakhs was covered by insurance. Everyone feels happy if they got cured, but now I don’t feel happy due to economic problems” (57 Years/ Male)*
	*Hospital has given concession but we had to buy medicines. We spent more than one lakh. We both were daily wage labourers. But for treatment we had to pledge silver ornaments, and gold ornaments and spent the money. A Doctor gave concession, otherwise we would have suffered a lot. He gave costly medicines at free of cost, we bought the less expensive medicines. Total expense would have gone up to 10 Lakhs… But they have made free. So, we are safe.* (38/ Female)
Loss of Job due to COVID/ Wage loss	*“Initially, I had back pain and fever so I took 3 days leave and informed my leave to HR properly. HR agreed to give leave. When I go after one month, they asked to get a fitness certificate from CMC hospital. Then I got a fitness certificate and submitted, they said, “I’ll call you later”. Whenever I go they said, “I’ll call you later”. They didn’t give me a job.”* (33/ Male/ X-Ray Technician)
	*“I was hospitalized for two months and three months at home rest, so totally 5 months. I was earning Rs.25,000 to Rs.30,000 per month, so around Rs. 1,50,000, I lost because of COVID and also, I had to pay for my medical expenses”* (41/ Male/ Xerox shop)

### Qualitative analysis

Common themes that emerged during interviews included emotional trauma of being ostracized during the COVID pandemic, ongoing physical disability after COVID, lost job opportunities and medical expenses further worsening already precarious financial conditions. Excerpts are available in Table 3.

## Discussion

Long COVID has now been described in various cohorts worldwide. The spectrum includes both persistence of clinical symptoms as well as an impairment of QoL in those infected. In most studies, there seems to be a correlation between development of severe acute COVID-19 and the subsequent development of long COVID [16]. Additionally, it has also been suggested that patients likely experienced higher levels of post-traumatic stress symptoms and depression due to novel nature of the disease and uncertainties regarding its progression, treatment and prevention [24,25].

Many studies with 3–6 months follow up after hospital discharge have been published [14–17,19,21] revealing a varied spectrum of post-discharge physical, emotional and mental characteristics [26]. Among the studies published in India, no studies have longitudinally assessed Quality of Life (QoL) at 1 year and hence our results indicating that almost 26% of patients continued to have challenges is quite significant. In other high- and middle-income countries, COVID-19 clinical symptoms were minimal for about 1–6 months after diagnosis, except in those with comorbidities [27,28]. Furthermore, poor QoL affecting physical and psychological aspects of well-being has also been reported even just one month after discharge [29,30].

Data is emerging that long COVID may persist up to 2 years [31,32]. In a metanalysis done by Cesar et al., [32] almost 30% of subjects who had been infected by SARS-CoV-2 continued to experience post-COVID symptoms beyond one year of acute COVID-19 infection with fatigue, cognitive disorders and pain being the most prevalent post-COVID symptoms within the general population.

Persistent muscle pain, muscle weakness, persistent joint pain and shortness of breath observed in our study were similar to results from a systematic review done with a minimum follow-up of one year which found that fatigue/weakness, dyspnea, arthromyalgia, depression, anxiety memory loss, concentration difficulties, and insomnia were the most prevalent symptoms ranging from 12 to 28% [33].

Our study suggested that persistence of shortness of breath, cough, joint pain at 6 weeks from hospital discharge for acute COVID-19, a history of ICU admission and prolonged hospital stay were predictors of long COVID, aligning with findings from another study by Naik Shivdas et al. [16] which stated that severity of COVID-19 infection (severe/moderate) seemed to correlate with development of long COVID. Persistent cough (8.6%) at 9–12 months of follow-up was a strong predictor of long COVID similar to findings from Bellan et al., who found cough and arthromyalgia, common in patients at 12 months [22,34].

Identifying predictors of long COVID is vital, as they help to prioritize the patients at risk and design interventions for appropriate management. This study provides a comprehensive overview of the symptoms reported by participants with long COVID-19 from 6 weeks after hospital discharge to 12 months after an acute COVID-19 infection. We also observed that persistence of more than two symptoms long-term had a significant impact on quality of life. In our study almost 35.2% of the participants still had more than 1 persistent symptom even at 6 weeks after hospital discharge which gradually reduced to 25.9% at 12 months.

The pathophysiology of long COVID is still being explored. A variety of pathophysiological mechanisms have been postulated including viral persistence with reduced viral cleavage leading to a persistent T cell response to SARS-CoV-2, oxidative stress leading to weak immune response and incomplete viral eradication, lower CD4 counts, increase in CD8 T cells, micro and macro vascular inflammation associated with increased levels of cytokines, circulating endothelial cells, coagulation activation as well as immune dysregulation [35,36].

Improving knowledge about pathophysiology indicates that biomarkers may also add value to early clinical prediction of long COVID and appropriate treatment strategies may be designed to reduce the incidence and prevalence of the same. Further studies are needed to confirm the validity of the physical and mental ailments as well as biological and radiological markers which could confirm presence of long COVID.

### Strengths and limitations

Our study’s strengths include its multi-centric design involving four tertiary care hospitals across different geographic regions. The main limitation of this study is that the research was entirely based on data collection through telephonic communication without any detailed clinical, psychological and physical examinations. In addition, this study did not correlate the different SARS Co-V2 variants with presence of long COVID and it also preceded the widespread implementation of the vaccine and hence whether this data would change if severity of disease is reduced remains to be determined.

### Conclusion

Prevalence of long COVID in our study was 16.5%. Predictors of long COVID at 1 year included severe COVID infection, shortness of breath, cough, joint pain, prolonged hospital stay of > 7 days and admission into ICU. These findings highlight the need for a long-term follow-up with tailored rehabilitation programs in affected patients.

## Supporting information

S1 File Tables S1-S6.
(DOCX)
