# Supplementary material for: Prevalence and predictors of long COVID at 1 year in a cohort of hospitalized patients: A multicentric qualitative and quantitative study
Source: PLoS One. 2025 Apr 11;20(4):e0320643. doi: 10.1371/journal.pone.0320643 (PMC11990506; doi:10.1371/journal.pone.0320643)
Supplement: S1 File — Tables S1-S6. (DOCX) [file pone.0320643.s001.docx]

**Table: S1 Hospitals participating in the study with follow-up data**

| **Hospital** | **6 Weeks** | **3-6**  **Months** | **6-9 Months** | **9-12 Months** |
| --- | --- | --- | --- | --- |
| Apollo Health City, Hyderabad | 24 (7.6) | 24 (7.8) | 25 (8.3) | 23 (8.3) |
| Believers Church Medical College Hospital, Tiruvalla | 88 (27.9) | 77 (25.0) | 88 (29.2) | 91 (32.7) |
| Christian Medical College, Vellore | 141 (44.8) | 146 (47.4) | 129 (42.9) | 118 (42.4) |
| New Bombay Hospital, Mumbai | 62 (19.7) | 61 (19.8) | 59 (19.6) | 46 (16.5) |
| **Total** | **315 (100.0)** | **308 (100.0)** | **301 (100)** | **278 (100)** |

**Table S2: Baseline characteristics (demography and co-morbidities) according to the Severity of COVID**

| **Characteristics** | **Total**  **(N = 315)** | **Mild**  **(N = 132)** | **Moderate**  **(N = 85)** | **Severe**  **(N = 98)** | **p value** |
| --- | --- | --- | --- | --- | --- |
| Age - Mean (SD) | 51.98 (14.62) | 53.34 (14.81) | 53.19 (15.24) | 49.09 (13.50) | 0.062 |
| Gender (Male) | 187 (59.4) | 69 (52.3) | 61 (71.8) | 57 (58.2) | **0.016** |
| Education (Educated) | 152 (48.3) | 66 (50.0) | 45 (52.9) | 41 (41.8) | 0.283 |
| Occupation (Employed) | 205 (65.1) | 82 (62.1) | 65 (76.5) | 58 (59.2) | **0.032** |
| Diabetes Mellitus | 122 (38.7) | 43 (32.6) | 43 (50.6) | 36 (36.7) | **0.026** |
| Hypertension | 116 (36.8) | 47 (35.6) | 38 (44.7) | 31 (31.6) | 0.175 |
| Chronic Cardiac Diseases | 37 (11.7) | 11 (8.3) | 17 (20.0) | 9 (9.2) | **0.021** |
| Chronic Pulmonary Diseases | 18 (5.7) | 7 (5.3) | 7 (8.2) | 4 (4.1) | 0.466 |
| **Notes:**   1. Data are presented as No. (%) unless otherwise indicated. P values are determined using the analysis of variance significant difference test for continuous variables (age) and the Chi-square test for categorical variables 2. Diabetes Mellitus, Hypertension, Cardiac Disease & Chronic Pulmonary Diseases are the major comorbidities found in our cohort. Hence, reported the above-mentioned comorbidities. 3. Severity – Asymptomatic patients were in small numbers, hence we included in the mild severity and similarly critical also included in the severe category. | | | | | |

**Table S3: Frequency of symptoms at each follow-up**

| **Symptoms** | **At 6 Weeks** | **3-6 Months** | **6-9 Months** | **9-12 Months** |
| --- | --- | --- | --- | --- |
| One Symptom / No symptoms | 204 (64.8) | 211 (68.5) | 221 (73.4) | 206 (74.1) |
| More than one Symptom | 111 (35.2) | 97 (31.5) | 80 (26.6) | 72 (25.9) |
| **Total** | **315 (100.0)** | **308 (100.0** | **301 (100.0)** | **278 (100.0)** |

**Table S4: Quality of life at baseline (6 weeks) in each category of severity**

| **Best describes your health** | **Mild**  **(N=132)** | **Moderate**  **(N= 85)** | **Severe**  **(N=98)** | **Total**  **(N=315)** |
| --- | --- | --- | --- | --- |
| **Usual Activities** | | | | |
| No problem | 113 (46.9) | 62 (25.7) | 66 (27.4) | 241 (100.0) |
| Slight problems | 13 (25.5) | 19 (37.3) | 19 (37.3) | 51 (100.0) |
| Moderate problems | 3 (18.8) | 4 (25.0) | 9 (56.3) | 16 (100.0) |
| Severe problems | 2 (33.3) | 0 (0.0) | 4 (66.7) | 6 (100.0) |
| Unable to do | 1 (100.0) | 0 (0.0) | 0 (0.0) | 1 (100.0) |
| **Anxiety / Depression** | | | | |
| Not anxious or depressed | 116 (44.1) | 71 (26.9) | 76 (28.8) | 263 (100.0) |
| Slightly anxious or depressed | 13 (35.1) | 11 (29.7) | 13 (35.1) | 37 (100.0) |
| Moderately anxious or depressed | 2 (14.3) | 3 (21.4) | 9 (64.3) | 14 (100.0) |
| Severely anxious or depressed | 1 (100.0) | 0 (0.0) | 0 (0.0) | 1 (100.0) |
| **Pain / Discomfort** | | | | |
| No pain or discomfort | 116 (44.3) | 76 (29.0) | 70 (26.7) | 262 (100.0) |
| Slight pain or discomfort | 14 (38.9) | 6 (16.7) | 16 (44.4) | 36 (100.0) |
| Moderate pain or discomfort | 1 (7.7) | 3 (23.1) | 9 (69.2) | 13 (100.0) |
| Severe pain or discomfort | 1 (25.5) | 0 | 3 (75.0) | 4 (100.0) |

**Table S5: Univariable Logistic Regression (association between Long COVID, demography, comorbidities and symptoms)**

| **Variables** | **Label** | **Long COVID** | | | **Univariable Logistic Regression** | |
| --- | --- | --- | --- | --- | --- | --- |
|  |  | **No Long COVID**  **(N = 263)** | **Long COVID**  **(N = 52)** | **Total**  **(N = 315)** | **Odds Ratio** | **p-value** |
| **Demographic** | | | | | | |
| Age | Less than 60 Years | 179 (68.06) | 40 (76.92) | 219 (69.52) | 1.56 (0.78-3.13) | 0.207 |
|  | Above 61 Years | 84 (31.94) | 12 (23.08) | 96 (30.48) | **Ref** |  |
| Gender | Male | 159 (60.46) | 25 (53.85) | 187 (59.37) | 0.76 (0.41-1.38) | 0.376 |
|  | Female | 104 (39.54) | 24 (46.15) | 128 (40.63) | **Ref** |  |
| Education | Up to School Education | 136 (51.71) | 27 (51.92) | 163 (51.75) | 1.46 (0.83-2.55) | 0.181 |
|  | Degree and Above | 127 (48.29) | 25 (48.08) | 152 (48.25) | **Ref** |  |
| Occupation | Unemployed | 89 (33.8) | 21 (40.4) | 110 (34.9) | 0.75 (0.41-1.38) | 0.367 |
|  | Employed | 174 (66.2) | 31 (59.6) | 205 (65.1) | **Ref** |  |
| **Comorbidities** | | | | | | |
| Cardiac Diseases | Yes | 32 (12.17) | 5 (9.62) | 37 (11.75) | 0.76 (0.28-2.07) | 0.602 |
|  | No | 231 (87.83) | 47 (90.38) | 278 (88.25) | Ref |  |
| Hypertension | Yes | 99 (37.64 | 17 (32.69) | 116 (36.83) | 0.80 (0.42-1.41) | 0.499 |
|  | No | 164 (62.36) | 35 (67.31) | 199 (63.17) | **Ref** |  |
| Diabetes | Yes | 101 (38.40) | 21 (40.38) | 122 (38.73) | 1.08 (0.59-1.99) | 0.789 |
|  | No | 162 (61.60) | 31 (59.62) | 193 (61.27) | **Ref** |  |
| Smoking | Yes | 11 (4.18) | 2 (3.85) | 13 (4.13) | 0.91 (0.19-4.26) | 0.911 |
|  | No | 252 (95.82) | 50 (96.15) | 302 (95.87) | **Ref** |  |
| Alcohol | Yes | 23 (8.75) | 3 (5.77) | 26 (8.25) | 0.63 (0.18-2.21) | 0.479 |
|  | No | 240 (91.25) | 49 (94.23) | 289 (91.75) | **Ref** |  |
| ICU | Yes | 56 (21.3) | 27 (51.9) | 83 (26.35) | 4.27 (2.27 – 8.0) | <0.001 |
|  | No | 204 (78.7) | 23 (48.1) | 227 (73.7) | **Ref** |  |
| Severity | Mild | 122 (46.4) | 10 (19.2) | 132 (41.9) | **Ref** | 0.001 |
|  | Severe | 141 (53.6) | 42 (80.8) | 183 (58.1) | 3.63 (1.74-7.54) |  |
| Hospital Stay | Less than 7 Days | 153 (58.2) | 16 (30.8) | 169 (53.7) | **Ref** | <0.001 |
|  | More than 7 Days | 110 (41.8) | 36 (69.2) | 146 (46.3) | 3.12 (1.65-0.17) |  |
| **Symptoms** | | | | | | |
| Headache | Yes | 21 (7.98) | 5 (9.62) | 26 (8.25) | 1.22 (0.44-3.41) | 0.697 |
|  | No | 242 (92.02) | 47 (90.38) | 289 (91.75) | **Ref** |  |
| Cough | Yes | 18 (6.84) | 12 (23.08) | 30 (9.52) | 4.08 (1.82-9.11) | 0.001 |
|  | No | 245 (93.16) | 40 (76.92) | 285 (90.48) | **Ref** |  |
| Shortness of Breath | Yes | 42 (15.97) | 21 (40.38) | 63 (20.0) | 3.56 (1.87-6.79) | <0.001 |
|  | No | 221 (84.03) | 31 (59.62) | 252 (80.0) | **Ref** |  |
| Pain on Breathing | Yes | 10 (3.80) | 6 (11.54) | 16 (5.08) | 3.3 (1.14-9.52) | 0.027 |
|  | No | 253 (96.20) | 46 (88.46) | 299 (94.92) | **Ref** |  |
| Chest Pain | Yes | 3 (1.14) | 4 (7.69) | 7 (2.22) | 7.22 (1.56-33.29) | 0.011 |
|  | No | 260 (98.86) | 48 (92.31) | 308 (97.78) | **Ref** |  |
| Palpitations | Yes | 4 (1.52) | 3 (5.77) | 7 (2.22) | 3.96 (0.86-18.26) | 0.07 |
|  | No | 259 (98.48) | 49 (94.23) | 308 (97.78) | **Ref** |  |
| Weight Loss | Yes | 32 (12.17) | 16 (30.77) | 48 (15.24) | 3.20 (1.60-6.43) | 0.001 |
|  | No | 231 (87.83) | 36 (69.23) | 267 (84.76) | **Ref** |  |
| Loss of Appetite | Yes | 6 (2.28) | 2 (3.85) | 8 (2.54) | 1.71 (0.33-8.73) | 0.517 |
|  | No | 257 (97.72) | 50 (96.15) | 307 (97.46) | **Ref** |  |
| Constipation | Yes | 6 (2.28) | 2 (3.85) | 8 (2.54) | 1.71 (0.33-8.73) | 0.517 |
|  | No | 257 (97.72) | 50 (96.15) | 307 (97.46) | **Ref** |  |
| Problems in balance | Yes | 10 (3.80) | 1 (1.92) | 11 (3.49) | 0.49 (0.06-3.96) | 0.508 |
|  | No | 253 (96.20) | 51 (98.08) | 304 (96.51) | **Ref** |  |
| Weakness in arms or legs / muscle weakness | Yes | 47 (17.87) | 11 (21.15) | 58 (18.41) | 1.23 (0.59-2.57) | 0.577 |
|  | No | 216 (82.13) | 41 (78.85) | 257 (81.59) | **Ref** |  |
| Persistent muscle pain | Yes | 9 (3.42) | 7 (13.46) | 16 (5.08) | 4.39 (1.55-12.38) | 0.005 |
|  | No | 254 (96.58) | 45 (86.54) | 299 (94.92) | **Ref** |  |
| Joint Pain | Yes | 15 (5.70) | 9 (17.31) | 24 (7.62) | 3.46 (1.42-8.40) | 0.006 |
|  | No | 248 (94.30) | 43 (82.69) | 291 (92.38) | **Ref** |  |
| Can’t move or control movements | Yes | 10 (3.80) | 1 (1.92) | 11 (3.49) | 0.49 (0.06-3.96) | 0.508 |
|  | No | 253 (96.20) | 51 (98.08) | 304 (96.51) | **Ref** |  |
| Confusion / lack of concentration | Yes | 12 (4.56) | 2 (3.85) | 14 (4.44) | 0.83 (0.18-3.85) | 0.819 |
|  | No | 251 (95.44) | 50 (96.15) | 301 (95.56) | **Ref** |  |
| Problems in Sleeping | Yes | 10 (3.80) | 5 (9.62) | 15 (4.76) | 2.69 (0.88-8.23) | 0.083 |
|  | No | 253 (96.20) | 47 (90.38) | 300 (95.24) | **Ref** |  |

**Table S6: COnsolidated criteria for REporting Qualitative research (COREQ) Checklist**

| **No.** | **Item** | **Description** | **Section#** |
| --- | --- | --- | --- |
| **Domain 1: Research team and reflexivity** | | | |
| **Personal characteristics** | | | |
|  | Interviewer/facilitator | Which author/s conducted the interview or focus group? | One of the investigators (JVK) conducted the in-depth interviews |
|  | Credentials | What were the researcher's credentials? E.g. PhD, | M.S.W., Ph.D., |
|  | Occupation | What was their occupation at the time of the study? | Social Scientist |
|  | Gender | Was the researcher male or female? | Male |
|  | Experience and  training | What experience or training did the researcher have? | The researcher has 10 years of research experience and published qualitative research papers as well.  JKV attended workshop on “Qualitative Research Methods & Analysis” |
| **Relationship with participants** | | | |
|  | Relationship  established | Was a relationship established prior to study commencement? | The interviewer (JVK) had no relationship with the participating group. Contacted only for the purposes of this research. |
|  | Participant knowledge  of the interviewer | What did the participants know about the researcher? E.g. Personal goals, reasons for doing the research | The researcher introduced himself to the participant. He assured the participants that the interviews were being conducted to understand the health problems, stigma and discrimination, financial burden due to COVID and loss of job and loss of wage due to COVID. |
|  | Interviewer  characteristics | What characteristics were reported about the interviewer/facilitator? E.g. Bias, assumptions, reasons and interests in the research topic | No bias was identified. |
| **Domain 2: Study design** | | | |
| **Theoretical framework** | | | |
|  | Methodological  orientation and theory | What methodological orientation was stated to underpin the study? E.g. grounded theory,  discourse analysis, ethnography,  phenomenology, content analysis | Grounded theory was chosen for this study because of its ability to develop concepts and models from information ‘grounded’ in the data, serving to explain the content of texts, rather than being based on a priori theory or assumption. |
|  | Sampling | How were participants selected? E.g. purposive, convenience, consecutive, snowball | Convenience Sampling was used |
|  | Method of approach | How were participants approached? E.g. face to-face, telephone, mail, email | All the in-depth interviews were conducted face to face. |
|  | Sample size | How many participants were in the study? | 13 interviews we conducted. 10 interviews were included. |
|  | Non-participation | How many people refused to participate or dropped out? What were the reasons for this? | 3 interviews were not included in the study as the patient was sick unable to complete the interviews. |
| **Setting** | | | |
|  | Setting of data  collection | Where was the data collected? E.g. home, clinic, workplace | The interviews were conducted at participant’s home and at the health facility. |
|  | Presence of nonparticipants | Was anyone else present besides the participants and researchers? | No |
|  | Description of sample | What are the important characteristics of the sample? E.g. demographic data, date | The participant’s important characteristics were reported along with the excerpts |
| **Data Collection** | | | |
|  | Interview guide | Were questions, prompts, guides provided by the authors? Was it pilot tested? | Interview guide was pre-tested with two patients before we administered the final one. |
|  | Repeat interviews | Were repeat interviews carried out? If yes, how many? | No repeat interviews were carried out. |
|  | Audio/visual recording | Did the research use audio or visual recording to collect the data? | All the interviews were audio recorded. |
|  | Field notes | Were field notes made during and/or after the interview or focus group? | No |
|  | Duration | What was the duration of the interviews or focus group? | The average duration of interviews were 17 – 20 minutes. |
|  | Data saturation | Was data saturation discussed? | Interviews were continuing until data saturation was reached. |
|  | Transcripts returned | Were transcripts returned to participants for comment and/or correction? | No |
| **Domain 3: analysis and findings** | | | |
|  | Number of data  coders | How many data coders coded the data? | Two investigators |
|  | Description of the  coding tree | Did authors provide a description of the coding tree? | Two researchers independently coded the data and discussed and resolved discrepancies in coding as needed. |
|  | Derivation of themes | Were themes identified in advance or derived  from the data? | Themes were derived from the data. |
|  | Software | What software, if applicable, was used to manage the data? | DEDOOSE Version 9.0.17  Los Angeles, CA: SocioCultural Research Consultants, LLC www.dedoose.com |
|  | Participant checking | Did participants provide feedback on the findings? | No |
| **Reporting** | | | |
|  | Quotations presented | Were participant quotations presented to illustrate the themes / findings? Was each  quotation identified? E.g. Participant number | Yes, each participants excerpt is presented in Table 3. |
|  | Data and findings  consistent | Was there consistency between the data presented and the findings? | Yes, the themes derived from the qualitative data are consistent with the quantitative data. |
|  | Clarity of major  themes | Were major themes clearly presented in the findings? | Yes, major themes were presented in the findings. |
|  | Clarity of minor  themes | Is there a description of diverse cases or discussion of minor themes? | Minor themes are not included. |
